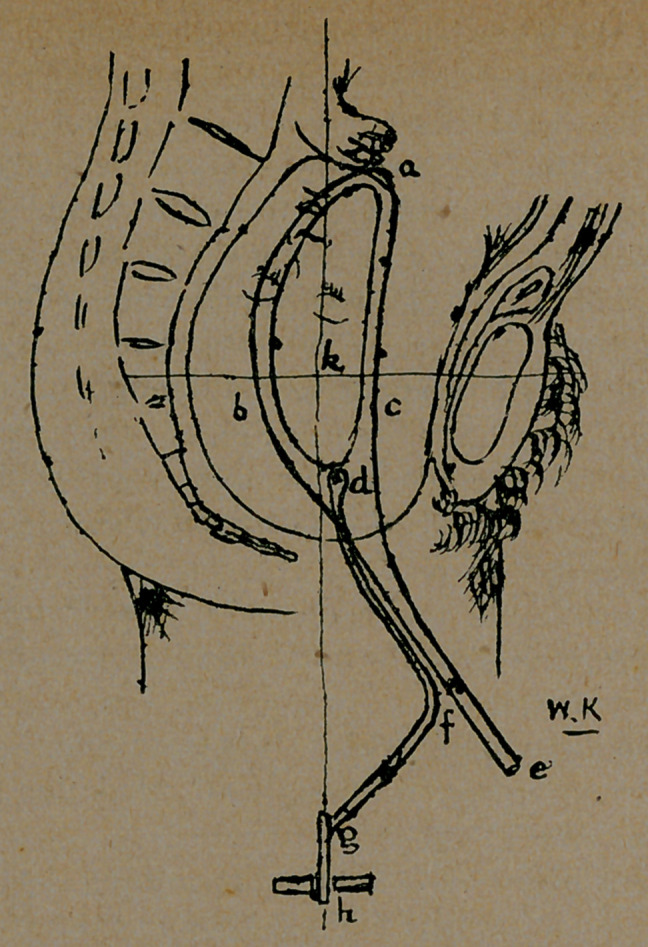# Axis Traction Forceps

**Published:** 1893-06

**Authors:** Wm. Keiller

**Affiliations:** M. D., of Edinburg, Professor of Anatomy, University Texas Medical College; S. S. Neuces, en route for New York, Atlantic Ocean


### AXIS TRACTION FORCEPS.

### Letter From Prof. Wm. Keiller, M. D., of Edinburg, Professor of Anatomy, University Texas Medical College.

S. S. NEUCES, EN ROUTE FOR NEW YORK,
ATLANTIC OCEAN, MAY, 15, 1893.

*Editor Daniel's Texas Medical Journal:*

A smooth sea, a blue sky, a cool breeze, and the somewhat limited society on this trip on the Mallory steamer are conditions conducive to thought; and my thoughts have wandered backward to, amongst other thimgs, my failure to send you my usual contributions to the JOURNAL. I trust, however, I may be able to send you one from Edinburgh for the July number.

Early in the past winter I read a paper at Galveston on Axis Traction Forceps, and though I was prepared to find them only in the hands of younger practitioners, I was not prepared to find them so completely absent from the local obstetrical armamenrium; still less was I prepared to find that in the exhibit of instruments at the Association meeting they should not be represented, or represented only by a pair of rods, to be adapted to ordinary long forceps, device familiar to the schools of long ago, and only indicating that the inventor is very imperfectly familiar with the principles of axis traction. I feel quite confident that no obstetrician who will once give them a fair trial and master the slight initial difficulty of their application, will ever use any other instrument. But many of the instruments at present in the market are by no means true axis traction forceps.

Let me explain shortly the principles of their construction and use, and for further details, let me refer your readers to my paper in the *American Journal of Obstetrics and Diseases of Women* for April of this year.

1. The parturient canal is curved from entrance to exit.

2. During its descent the fœtal head is constantly changing in three different ways.

a. In the direction in which it is travelling, i. e., first downwards and backwards, and through all gradation to downwards and forwards.

b. In the amount of flexion of the chin on the sternum.

c. The presenting part tends to rotate to the front or rear.

From these combined facts a true traction must answer four conditions.

1st. It must be capable of producing traction in the exact direction of that part of the parturient canal through which the fœtal head is traveling without interference with the constant change in direction which the head must adapt itself to.

2nd. *It must indicate the exact direction in which traction should be applied.*

3rd. It must not interfere with the flexion of the head on the sternum.

4th. It must admit of rotation of the presenting part to the front or rear.

Ordinary long, and to a less extent short forceps, interfere with all these movements, are incapable in the most skilled hands of admitting traction in the right direction, and some experienced operators have a very imperfect idea of the direction in which they ought to draw down the child's head.

True axis forceps fulfill all these indications, and are ideal in their perfection.

a b c is the blade which grasps, without appreciably compressing, the child's head; d e, the shanks which serve two purposes. 1st. They are used for applying the blades and locking them. 2nd. They are a constant indicator of the direction in which traction is to be applied. *They must never be themselves used to pull with*; d f g the traction rods, jointed at d as nearly as possible to the centre of the head, and admitting of free flexion and extension of the head of the child. From their attachment at d, a point rather in front of the centre k of the fœtal head, they tend to favor rather then hinder flexion. They are so bent *that, when the portion of d f is parallel with the shanks*, the traction handle h is directly in the axis of the segment of the pelvis through which the head is passing. Thus as traction is applied, the shanks d e of the forceps move forward as the head descends, and if the operator will keep *following* the *shanks*, (not pushing them) with the rods, so as always to keep the portion d f parallel to the shanks *he cannot fail to pull in the right direction.* Lastly the joint at h admits of free axial rotation of the head to front or rear.

A word as to their indications. Short forceps are unnecessary. One pair of long axis traction forceps will do all that forceps can do. They are the best instruments, either at the brim or outlet.

Lacerations or rents, where they are properly used, should be the exception, not the rule.

With axis traction forceps a normal child can be delivered through a true conjugate of three inches.

With the addition of symphisiotomy it can be delivered through a true conjugate of 2½ inches.

Below that, the choice is between the indication of premature labor, cæsarian section and embryotomy.

I have been asked by students in the obstetrical clinic at Edinburg why the neck of a child can stand so much dragging. It is entirely a misconception to imagine that there is any appreciable *dragging* on the fœtal neck when the forceps act as a *wedge* and the uterus itself forces down the body of the child as fast as the head descends, the *wedge action of the blade* preparing the way. I have twice had the forceps on the child's head for two hours without doing it any damage whatever.

I trust I have succeeded in again drawing the attention of my professional brethren with some success to this important addition to the obstetrical outfit, and permit me for once to sign myself in view of recently past events. Sincerely yours,

* "THE GENTLEMAN FROM THE LAND O' CAKES."

*In debate at Galveston our facetious friend, Dr. Q. C. Smith alluded to Prof. Keiller "as the gentleman from the land o' cakes and ale."

## Figures and Tables

**Figure f1:**